# Mechanism of the efflux transport of demethoxycurcumin-*O*-glucuronides in HeLa cells stably transfected with UDP-glucuronosyltransferase 1A1

**DOI:** 10.1371/journal.pone.0217695

**Published:** 2019-05-31

**Authors:** Beibei Zhang, Jing Yang, Zifei Qin, Shishi Li, Jinjin Xu, Zhihong Yao, Xiaojian Zhang, Frank J. Gonzalez, Xinsheng Yao

**Affiliations:** 1 Department of Pharmacy, the First Affiliated Hospital of Zhengzhou University, Zhengzhou, China; 2 College of Pharmacy, Jinan University, Guangzhou, China; 3 Guangdong Provincial Key Laboratory of Pharmacodynamic Constituents of TCM and New Drugs Research, Jinan University, Guangzhou, China; 4 Laboratory of Metabolism, Center for Cancer Research, National Cancer Institute, National Institutes of Health, Bethesda, Maryland, United States of America; Institute of Biochemistry and Biotechnology, TAIWAN

## Abstract

Demethoxycurcumin (DMC) is a safe and natural food-coloring additive, as well as an agent with several therapeutic properties. However, extensive glucuronidation *in vivo* has resulted in its poor bioavailability. In this study, we aimed to investigate the formation of DMC-*O*-glucuronides by uridine 5'-diphospho-glucuronosyltransferase 1A1 (UGT1A1) and its transport by breast cancer resistance protein (BCRP) and multidrug resistance-associated proteins (MRPs) in HeLa cells stably transfected with UGT1A1 (named HeLa1A1 cells). The chemical inhibitors Ko143 (a selective BCRP inhibitor) and MK571 (a pan-MRP inhibitor) both induced an obvious decrease in the excretion rate of DMC-*O*-glucuronides and a significant increase in intracellular DMC-*O*-glucuronide concentrations. Furthermore, BCRP knock-down resulted in a marked reduction in the level of excreted DMC-*O*-glucuronides (maximal 55.6%), whereas MRP1 and MRP4 silencing significantly decreased the levels of excreted DMC-*O*-glucuronides (a maximum of 42.9% for MRP1 and a maximum of 29.9% for MRP3), respectively. In contrast, neither the levels of excreted DMC-*O*-glucuronides nor the accumulation of DMC-*O*-glucuronides were significantly altered in the MRP4 knock-down HeLa cells. The BCRP, MRP1 and MRP3 transporters were identified as the most important contributors to the excretion of DMC-*O*-glucuronides. These results may significantly contribute to improving our understanding of mechanisms underlying the cellular disposition of DMC *via* UGT-mediated metabolism.

## Introduction

Demethoxycurcumin (DMC) is one of the most abundant curcuminoids present in turmeric (*Curcuma longa*) and accounts for 0.03 to 9.26% of the dry weight of the rhizome (mg/g) [1]. As a curcumin analog, DMC has attracted increasing attention, particularly regarding its biological activities. For instance, the combination of gefitinib with DMC may potentially be used as an anticancer agent for human oral cancer in the future [2]. In addition, DMC is a promising antioxidant and anti-inflammatory agent [3]. DMC displays promising therapeutic potential as a treatment for Alzheimer's disease [4]. Furthermore, DMC possesses unique molecular activities, including the induction of androgen receptor (AR) degradation and the suppression of the transcription factor activator protein-1 (AP-1) [5]. Moreover, DMC may also reduce cyclooxygenase-2 (COX-2) expression and improve endothelial function in subjects with hypertension [6]. These remarkable pharmacological properties are known to be severely limited by the low bioavailability of DMC, which results from the poor absorption and extensive metabolism of this compound.

Since considerable interest in these beneficial effects of DMC on human health exists, the pharmacokinetics and metabolism of orally delivered DMC have been well characterized. For example, when 2 g of curcuminoids, including 12% DMC, were orally administered to two healthy volunteers, the DMC concentration was below the detection limit (2.0 ng/mL) [7]. Similar results were also observed in equine plasma [8] and mouse plasma [9]. A potential explanation for the undetectable level of DMC is that oral administration results in the excretion of most of the DMC and curcumin in the feces and to a lesser extent in the urine [10]. In addition, the extensive metabolism of DMC *in vivo* and its poor absorption are other important factors that affect the oral bioavailability of DMC. For instance, nine phase I metabolites were isolated and identified based on nuclear magnetic resonance (NMR) data after the oral administration of DMC (50 mg/kg) [11]. Demethylation is also a major pathway of DMC metabolism by the human intestinal bacterium *Blautia* sp. MRG-PMF1 [12]. Moreover, glucuronide and sulfate conjugates were identified as the most abundant phase I metabolites of DMC in rat plasma (approximately 50 nM) [13,14].

Several scholars have expended substantial efforts on enhancing the oral bioavailability of DMC by altering its preparation to address these issues. After treatment with DMC-polymeric *N*-isopropyl acryl amide (PNIPAM) nanoparticles (i.n., 0.1 mg/kg), the C_max_ and AUC_0-t_ values increased 7-fold and 15-fold, respectively, compared with DMC [15]. These findings were consistent with a study of healthy humans who received a single oral dose of 98 mg of curcuminoid micelles or curcuminoid plus phytochemical micelles [16]. In another study, the plasma bioavailability, *in vivo* stability and blood-brain barrier permeability were all significantly increased (*p* < 0.001), as evidenced by the tissue distribution of free DMC in the liver, kidney, heart, spleen, and intestine after treatments with approximately 120, 80, 140, 50, and 150000 ng/g DMC, with an extended elimination half-life of 3 to 4 h [17]. Although the preparation design improved the absorption of DMC, extensive first-pass metabolism, particularly glucuronidation, is still the major reason for the poor bioavailability in animals and humans, which limited the uses of DMC as a therapeutic agent.

The glucuronidation of DMC, which results from a conjugation reaction with glucuronic acid, has been extensively identified in humans [18]. Uridine 5'-diphospho-glucuronosyltransferase 1A1 (UGT1A1) displays the most efficient catalytic activities (approximately 1600 pmol/min/mg) compared with UGT1A3, 1A8, 1A10 and 2B7 (less than 1100 pmol/min/mg) [18]. In general, drug elimination *via* the glucuronidation pathway involves at least two distinct and sequential processes, namely, glucuronide formation and excretion [19]. Because glucuronides are impermeable to cell membranes due to their high hydrophilicity, active transport of glucuronides by efflux transporters, primarily including breast cancer resistance protein (BCRP) and multidrug resistance-associated proteins (MRPs), is necessary [19]. Moreover, efflux transporters appear to function in concert with UGT enzymes to efficiently remove drugs from the body, which is a phenomenon termed glucuronidation-transport interplay; this phenomenon also plays a critical role in determining the oral bioavailability and pharmacokinetics of drugs undergoing glucuronidation [20,21]. However, drug disposition and excretion *via* efflux transporters has been poorly characterized. Therefore, we aimed to investigate the mechanisms of DMC disposition *via* glucuronide formation and excretion.

As described in our previous study [21], a HeLa cell line stably overexpressing UGT1A1 was established and successfully applied to evaluate the roles of BCRP and MRPs in the excretion of wushanicaritin-*O*-glucuronides. Similarly, two independent experiments, including the use of chemical inhibitors and short hairpin RNA-mediated silencing of the BCRP and MRPs transporters, were performed to identify active glucuronide transporters. These results will be valuable for achieving better predictions of DMC disposition, which may be the main factor affecting its bioavailability and biological activities. In addition, these findings will also improve our general understanding of the metabolic fate of DMC *in vivo*. Moreover, this cell model represented a practical and feasible approach to evaluate the UGT-catalyzed glucuronidation and efflux transport-mediated excretion of clinical drugs and xenobiotics.

## Materials and methods

### Materials and reagents

Demethoxycurcumin (DMC) was provided by Guangzhou Fans Biotechnology Co., Ltd. (Guangzhou, China). Alamethicin, D-saccharic-1,4-lactone, magnesium chloride (MgCl_2_), MK571, Ko143, propofol, and uridine diphosphate glucuronic acid (UDPGA) were purchased from Sigma-Aldrich (St. Louis, MO). Human UGT1A1 and individual human liver microsomes (iHLM, n = 12) were obtained from Corning Biosciences (New York, USA). UGT1A1-overexpressing HeLa cells (HeLa1A1 cells) were established as described in a previous study [22] and provided by Prof. Baojian Wu, who works in Jinan University in Guangzhou of China. β-Estradiol and β-estradiol-3-*O*-glucuronide were purchased from Toronto Research Chemicals (North York, ON, Canada). The anti-UGT1A1 antibody was purchased from BD Biosciences (Woburn, MA). The anti-BCRP (catalog number TA322704), anti-MRP1 (catalog number TA309559), anti-MRP2 (catalog number TA313641), anti-MRP3 (catalog number TA314800), and anti-MRP4 (catalog number TA327332) antibodies were purchased from OriGene Technologies (Rockville, MD). All other chemicals and reagents (analytical grade or better) were commercially available.

### HeLa1A1 cell culture

HeLa1A1 cells were seeded into six-well plates at a density of 4.0 × 10^5^ cells/well and cultured in Dulbecco’s Modified Eagle’s Medium (DMEM) supplemented with 10% fetal bovine serum (FBS). At 48 h after seeding, cells were used for the glucuronide excretion experiments. A detailed characterization of our stable UGT1A1-expressing HeLa cells was provided in previous studies [21,22]. The numbers of cells correlated with the protein concentrations of the harvested cells, which were determined with a protein assay kit (Bio-Rad, Hercules, CA) using bovine serum albumin as the standard. The HeLa cell volume was estimated to be 4 μL/mg protein to determine the intracellular glucuronide concentrations [23].

### Excretion experiments

Prior to the assays, HeLa1A1 cells were washed twice with prewarmed (37°C) Hank’s balanced salt solution (HBSS, pH = 7.4). Subsequently, HeLa1A1 cells were pretreated and incubated with DMC (2, 4 or 8 μM) and dissolved in 2 mL of HBSS. The sampling times were selected to ensure that the plots of the amount excreted *versus* time remained in the linear range. At each time point (0.5, 1.0, 1.5 and 2.0 h), 200 μL of incubation solutions were collected from each well, and an equal volume of loading media was used to replenish each well. Then, the collected samples were each mixed with 100 μL of ice-cold acetonitrile. The supernatants (8.0 μL) were subjected to an ultra high-performance liquid chromatography (UHPLC) analysis after centrifugation (10 min at 13,800 *g*).

Furthermore, the excretion rates of generated glucuronides were calculated using Eq (1). In addition, the fraction metabolized (*f*_met_) value was defined as the fraction of the dose metabolized based on Eq (2). *V* represents the volume of the incubation medium, C represents the concentration of excreted glucuronides, and t represents the incubation time.

$$\mathrm{Excretion}\;\mathrm{rate}=V\frac{\mathrm{dC}(\mathrm{excreted}\;\mathrm{glucuronide})}{\mathrm{dt}}$$ (1)

$$f_{met}=\frac{excreted\;glucuronide+intracellular\;glucuronide}{dosed\;aglycone}$$ (2)

### Functional validation in HeLa1A1 cells

Because β-estradiol is the probe substrate of UGT1A1 [24], β-estradiol-3-*O*-glucuronidation has been widely used to identify the function of UGT1A1. Similarly, we validated the function of this enzyme in HeLa1A1 cells by assessing β-estradiol-3-*O*-glucuronidation. β-Estradiol (5 and 20 μM) was dissolved in 2 mL of HBSS and incubated with HeLa1A1 cells. After the excretion experiments, the excreted β-estradiol-3-*O*-glucuronide concentrations and related excretion rates were determined using UHPLC.

### Preparation of HeLa1A1 cell lysates

HeLa1A1 cells were grown in 10-cm dishes for 72 h and then washed and harvested in 50 mM Tris buffer (pH 7.4). The collected cells were sonicated using a tight-fitting Dounce homogenizer in an ice-cold water bath (4°C). Subsequently, the cell lysates were centrifuged at 13,800 x *g* for 10 min (4°C). The supernatant was collected for use in the UGT glucuronidation activity assay. The protein concentration was determined using the bicinchoninic acid (BCA) assay (Beyotime, Shanghai, China). Due to the thermal stability of UGT1A1, the glucuronidation activity of UGT1A1 was not affected during sonication process.

### Glucuronidation activity assays

The glucuronidation assay was performed as previously described [25]. Briefly, alamethicin (22 μg/mL), D-saccharic-1,4-lactone (4.4 mM), MgCl_2_ (0.88 mM), UGT1A1 (1.0 mg/mL) or HeLa1A1 cell lysates (2.3 mg/mL) and DMC (0.5 to 40 μM) were mixed in a 50 mM Tris buffer (pH 7.4). After a preincubation at 37°C for 5 min, UDPGA (3.5 mM) was added to the incubation system, and the mixture was further incubated. After 30 min, the reactions were terminated by the addition of ice-cold acetonitrile (200 μL). The mixed samples were centrifuged at 13,800 x *g* for 10 min, and the supernatant was analyzed using UHPLC.

The Michaelis-Menten equation was fitted to the data for metabolic rates *versus* substrate concentrations, as displayed in Eq (3). The best model was selected based on a visual inspection of the Eadie-Hofstee plots [26]. Briefly, the rates (*V*) of glucuronide formation at various substrate concentrations (*S*) were fitted based on the standard Eq (3). K_m_ represents the Michaelis-Menten constant and *V*_max_ represents the maximum rate of glucuronidation. The intrinsic clearance (CL_int_) was derived from *V*_max_/K_m_. Model fitting and parameter estimation were performed by GraphPad Prism V5 software (SanDiego, CA, USA).

$$V=\frac{V_{max}\times [S]}{K_{m}+[S]}$$ (3)

### Analyses of chemical inhibition and activity correlations

Analyses of chemical inhibition and activity correlations were performed as two additional independent assays to determine the roles of the UGT1A1 enzyme [27,28]. In these studies, nilotinib (10 μM), glycyrrhetinic acid (20 μM) and protopanaxatriol (500 μM) were used as UGT1A1 inhibitors to investigate the metabolic activities of DMC (4 μM) [27,28]. The incubation conditions were the same as those used for the glucuronidation activity assays.

In addition, according to the glucuronidation assay protocol described in previous studies [24,25], the glucuronidation activities of iHLM (n = 12) toward DMC (4 μM) and a probe substrate for UGT1A1, β-estradiol (50 μM), were determined. Furthermore, a correlation analysis was performed between DMC-*O*-glucuronidation (G1 and G2) and β-estradiol-3-*O*-glucuronidation. The correlation (Pearson) analysis was performed using GraphPad Prism V5 software (SanDiego, CA, USA).

### Effects of Ko143 and MK571 on excretion

Ko143 and MK571 are well-accepted chemical inhibitors of BCRP and MRPs, respectively [21,22]. In this study, Ko143 (5 and 20 μM) and MK571 (5 and 20 μM) were separately dissolved in HBSS containing DMC (4 μM) to investigate their effects on the efflux transporters BCRP and MRPs, respectively. During the excretion experiments, DMC (4 μM) did not exert significant toxicity toward HeLa1A1 cells at the experimental concentration, enabling sufficient evaluation of glucuronide excretion.

### Effects of Ko143 and MK571 on glucuronidation activity

Ko143 (5 and 20 μM) and MK571 (5 and 20 μM) were separately included in the incubation mixture, as reported in a previous study [21], to obtain a better understanding of the effects of specific transporter inhibitors (Ko143 and MK571) on the glucuronidation activity of DMC. The metabolic rates (DMC-*O*-glucuronidation) were compared with the control group.

### Establishment of HeLa1A1-BCRP or MRP-shRNA cells

The shRNA plasmids targeting the efflux transporters BCRP, MRP1, MRP3 and MRP4 were constructed using previously described methods [29]. HeLa1A1 cells were transiently transfected with the corresponding shRNA plasmids. The procedures for the establishment of HeLa1A1-BCRP-shRNA cells, HeLa1A1-MRP1-shRNA cells, HeLa1A1-MRP3-shRNA cells and HeLa1A1-MRP4-shRNA cells are detailed in our previous publication [22]. Moreover, levels of the BCRP, MRP1, MRP3 and MRP4 proteins in these constructed HeLa1A1 cells were determined by western blotting assays, as described previously [22].

### Western blotting assays

The experimental procedure for western blotting was similar to a previously published protocol [22]. Briefly, HeLa1A1 cell lysates (40 μg of total proteins) were separated by 8% sodium dodecyl sulfate (SDS)-polyacrylamide gel electrophoresis and transferred onto polyvinylidene difluoride membranes (Millipore, Bedford, MA). Blots were probed with anti-UGT1A1, anti-BCRP, anti-MRP1, anti-MRP2, anti-MRP3, and anti-MRP4 antibodies, followed by horseradish peroxidase-conjugated rabbit anti-goat IgG (Santa Cruz Biotechnology, Santa Cruz, CA, USA). Protein bands were detected by enhanced chemiluminescence (ECL), and band intensities were measured by densitometry using the Quantity One software (Hercules, CA, USA).

### Quantification of DMC and DMC-*O*-glucuronide concentrations

DMC-*O*-glucuronide concentrations were quantified using an Acquity UHPLC I-Class system (Waters Corporation, Manchester, U.K.) equipped with a BEH C18 column (2.1 mm × 50 mm, 1.7 μm, Waters, Ireland, Part NO. 186002350) at 35°C. DMC and DMC-*O*-glucuronides were separated using water and acetonitrile (both including 0.1% formic acid, V/V) as the mobile phase at a rate of 0.4 mL/min. The following gradient elution program was used: 5% B from 0 to 0.5 min, 5 to 60% B from 0.5 to 1.7 min, 60% B from 1.7 to 2.0 min, and 60 to 90% B from 2.0 to 2.8 min, maintaining 90% B from 2.8 to 3.0 min, 90 to 5% B from 3.0 to 3.5 min, and maintaining 5% B from 3.5 to 4.0 min. The detection wavelength was 338 nm.

The UHPLC system was coupled to a hybrid quadrupole orthogonal time-of-flight tandem mass spectrometer (SYNAPT G2 HDMS, Waters, Manchester, U.K.) with electrospray ionization (ESI) mode. The following operating parameters were used: capillary voltage, 3 kV (ESI+); sample cone voltage, 35 V; extraction cone voltage, 4 V; source temperature, 100°C; desolvation temperature, 300°C; cone gas flow, 50 L/h and desolvation gas flow, 800 L/h. The full scan mass range was 50 to 1500 Da. Lock spray with leucine enkephalin (*m/z* 556.2771 in positive ion mode) was employed to ensure mass accuracy.

### Statistical analysis

All experiments were performed in triplicate (n = 3). The assay data are presented as means ± SD (n = 3). Mean differences between the treatment and control groups were analyzed using Student’s t test, whereas a one-way ANOVA was performed when data from more than two groups were compared by GraphPad Prism V5 software (SanDiego, CA, USA). The level of significance was set to *p* < 0.05 (*), *p* < 0.01 (**) or *p* < 0.001 (***).

## Results

### Functional validation of HeLa1A1 cells

First, β-estradiol, a probe substrate for the UGT1A1 enzyme [24], was used to confirm the overexpression of the UGT1A1 enzyme in HeLa1A1 cells. β-Estradiol-3-*O*-glucuronide, which is 176.032 Da larger than β-estradiol, was clearly produced after the incubation of β-estradiol with HeLa1A1 cells, as published in a recent study [30]. Furthermore, β-estradiol-3-*O*-glucuronide was excreted into the extracellular medium after the incubation of β-estradiol (5 and 20 μM) with HeLa1A1 cells and displayed a linear increase within 60 min, with excretion rates of 0.74 and 1.66 pmol/min after an incubation with 5 μM and 20 μM β-estradiol, respectively [30]. Moreover, these established HeLa1A1 cells were rather active in generating the glucuronides after incubations with wushanicaritin, chrysin, genistein, apigenin and other compounds [20–22].

### Generation of DMC-*O*-glucuronides in HeLa1A1 cells

**Fig 1:**
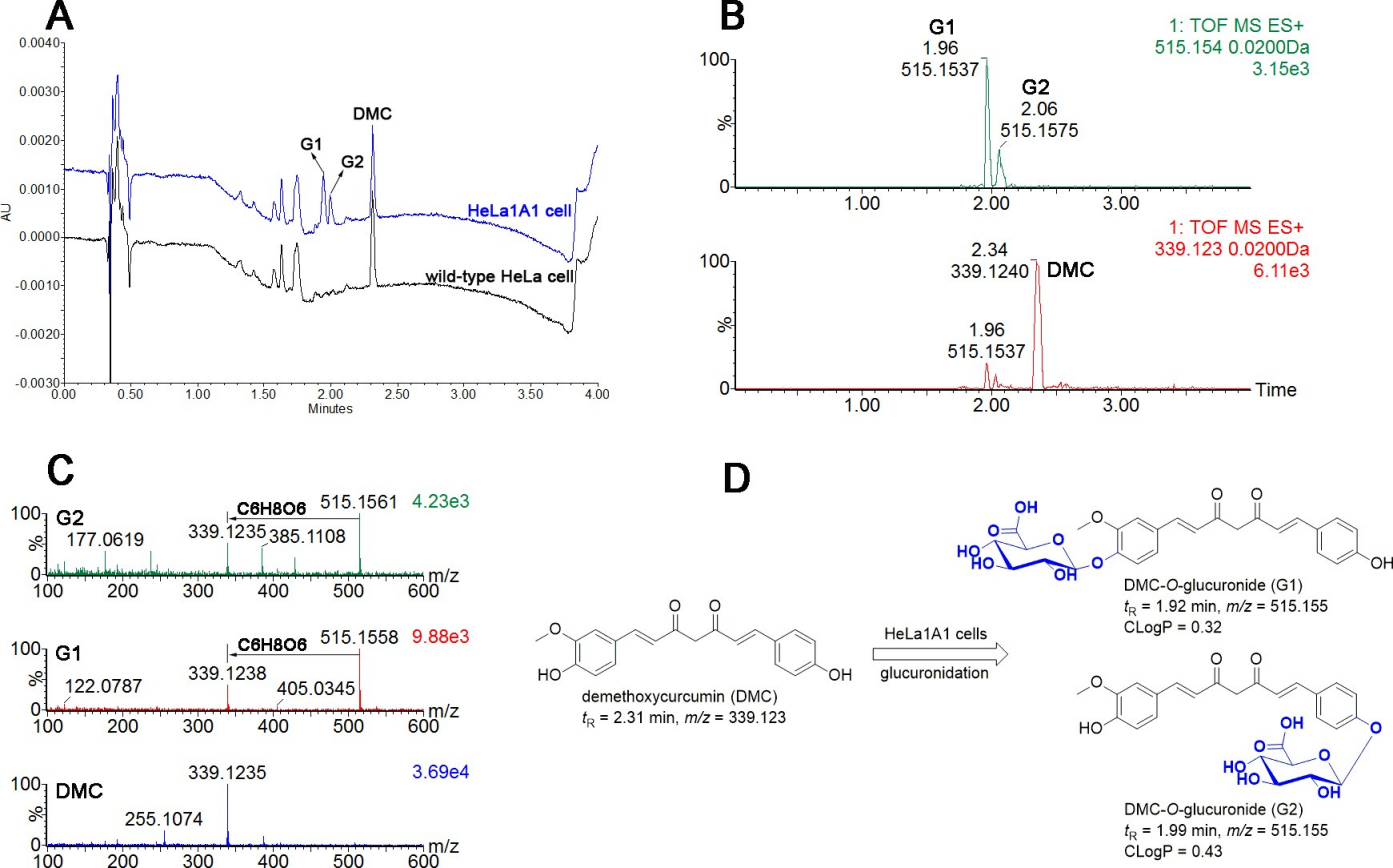
Liquid chromatography tandem mass spectrometry analysis of demethoxycurcumin and demethoxycurcumin-*O*-glucuronides. (A) Ultra high-performance liquid chromatography chromatograms of demethoxycurcumin and demethoxycurcumin-*O*-glucuronides (G1 and G2) generated in wild-type HeLa cells and HeLa1A1 cells; (B) Extracted ion chromatograms of demethoxycurcumin and demethoxycurcumin-*O*-glucuronides (G1 and G2); (C) ESI-MS/MS spectra of demethoxycurcumin and demethoxycurcumin-*O*-glucuronides (G1 and G2) in positive ion mode; (D) Glucuronidation pathways of demethoxycurcumin by HeLa1A1 cells.

After an incubation of HeLa1A1 cells or wild-type HeLa cells with DMC (4 μM), two obvious additional metabolites were detected in the HeLa1A1 cell incubation medium, whereas no metabolites were detected in the medium of the wild-type HeLa cells (Fig 1A). The extracted ion chromatograms (Fig 1B) and (+) ESI-MS/MS spectra (Fig 1C) indicated that these two metabolites were mono-glucuronidated DMC. Furthermore, according to the ClogP values, the metabolites were designated as **G1** (CLogP = 0.32) and **G2** (CLogP = 0.43), respectively (Fig 1D). Based on these results, HeLa1A1 cells were fairly active in generating and excreting DMC-*O*-glucuronides.

### DMC-*O*-glucuronidation activity of UGT1A1 and the HeLa1A1 cell lysate

**Fig 2:**
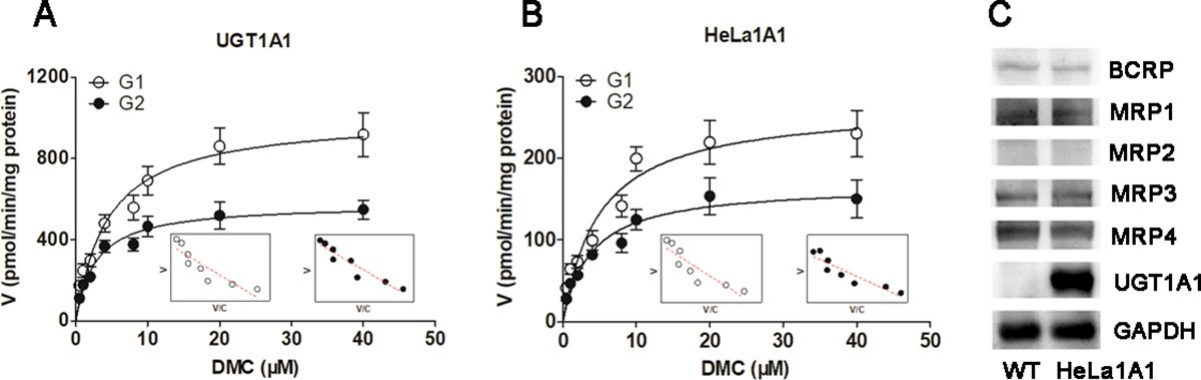
UGT1A1 catalyzed the demethoxycurcumin-*O*-glucuronidation, and expression of UGT1A1 and efflux transporters in HeLa1A1 cells. (A) Kinetic profiles for demethoxycurcumin-*O*-glucuronides (G1 and G2) (0.5–40 μM) by UGT1A1; (B) Kinetic profiles for demethoxycurcumin-*O*-glucuronides (G1 and G2) (0.5–40 μM) by HeLa1A1 cell lysate; In each panel, the insert figure showed the corresponding Eadie-Hofstee plot. (C) Protein expression of UGT1A1, BCRP and four MRP family transporters in HeLa and HeLa1A1 cells. All experiments were performed in triplicate (n = 3). Data were expressed as mean ± SD (n = 3).

The DMC-*O*-glucuronidation activity (G1 and G2) of the UGT1A1 enzyme (Fig 2A) and the HeLa1A1 cell lysate (Fig 2B) both followed the Michaelis-Menten equation. The K_m_ values for G1 glucuronidation by UGT1A1 or the HeLa1A1 cell lysate were not significantly different (*p* > 0.05) (Table 1), whereas obvious differences (*p* < 0.001) were observed in the *V*_max_ and *CL*_int_ values (Table 1). Similar results were obtained for the parameters of G2 (Table 1). An explanation for this finding may be that UGT1A1 was much more concentrated in the recombinant material than in the HeLa1A1 cell lysate preparation.

**Table 1:** Kinetic parameters derived from DMC-*O*-glucuronidation by UGT1A1 and HeLa1A1 cell lysate.

Subs.	Meta.	Enzyme	*V*_max_ (pmol/min/mg)	*K*_m_ (μM)	*CL*_int_ (μL/min/mg)	Model
DMC	G1	UGT1A1	1006.0±62.8	4.5±0.9	225.9±47.3	MM
DMC	G2	UGT1A1	576.1±27.0	2.7±0.5	212.6±38.3	MM
DMC	G1	HeLa1A1 cell	264.5±22.3 (***)	5.0±1.3	53.0±14.5 (***)	MM
DMC	G2	HeLa1A1 cell	167.4±11.3 (^###^)	3.8±0.9	43.9±10.4 (^###^)	MM

All experiments were supplemented with UDPGA, and performed in triplicate (n = 3). Data are presented as means ± SD. Subs., substrate; Meta., metabolite; *V*_max_, the maximal velocity; *K*_m_, the Michaelis constant; *CL*_int_, the intrinsic clearance. MM, Michaelis-Menten model.

*^, #^ *p* < 0.05

**^, ##^ *p* < 0.01 or

***^, ###^ *p* < 0.001 compared with the parameters of G1 and G2 produced by the UGT1A1 enzyme, respectively.

Furthermore, western blotting results showed high levels of the UGT1A1 protein in HeLa1A1 cells, whereas UGT1A1 was not expressed in wild-type HeLa cells (Fig 2C). Moreover, BCRP, MRP1, MRP3, and MRP4 were detected in both wild-type HeLa and HeLa1A1 cells, whereas MRP2 was not detected (Fig 2C). Notably, the wild-type and engineered HeLa1A1 cells exhibited an identical pattern of transporter expression (Fig 2C). Thus, the engineered HeLa1A1 cells expressed a significant amount of the active UGT1A1 protein.

### Role of UGT1A1 in DMC-*O*-glucuronidation

**Fig 3:**
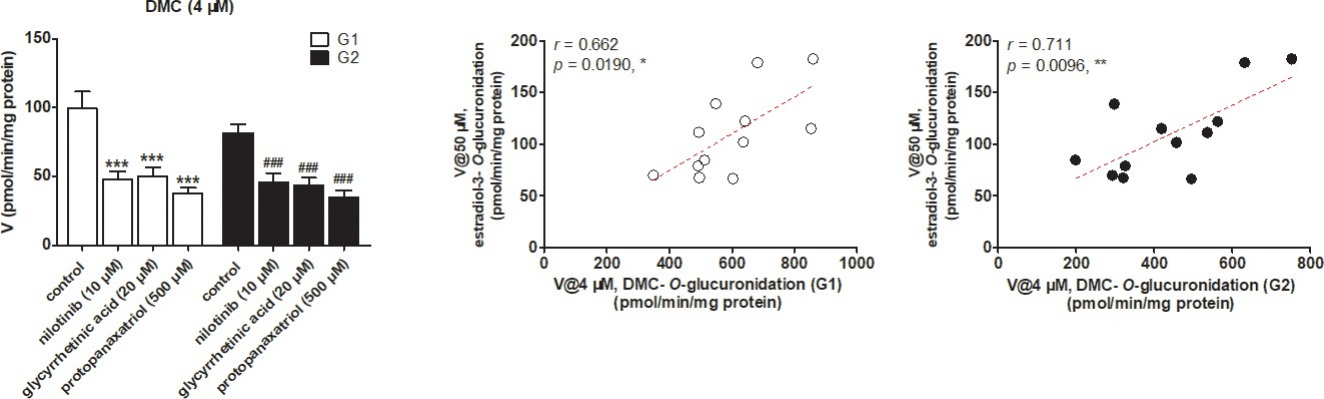
Validation of UGT1A1 in the demethoxycurcumin-*O*-glucuronidation by inhibitory assays and activity correlation analysis assays. (A) Inhibitory effects of nilotinib (10 μM), glycyrrhetinic acid (20 μM) and protopanaxatriol (500 μM) on the glucuronidation activities of G1 and G2 at 4 μM of demethoxycurcumin; (B) Correlation analysis between β-estradiol-3-*O*-glucuronidation and demethoxycurcumin-*O*-glucuronidation (G1) in a bank of individual human liver microsomes (n = 12); (C) Correlation analysis between β-estradiol-3-*O*-glucuronidation and demethoxycurcumin-*O*-glucuronidation (G2) in twelve individual HLMs; All experiments were performed in triplicate (n = 3). Data were expressed as mean ± SD (n = 3). *^, #^ *p* < 0.05, **^, ##^ *p* < 0.01 or ***^, ###^ *p* < 0.001 compared with that of control group of G1 and G2, respectively.

Chemical inhibitors were used to investigate the rates of G1 and G2 formation and to confirm the contribution of UGT1A1 to the generation of G1 and G2. Nilotinib (10 μM), glycyrrhetinic acid (20 μM) and protopanaxatriol (500 μM) all significantly inhibited the formation of G1 and G2 when the incubation mixture included DMC (4 μM) and the HeLa1A1 cell lysate (2.3 mg/mL) (Fig 3A).

In addition, DMC-*O*-glucuronidation (G1) was significantly correlated with β-estradiol-3-*O*-glucuronidation (with correlation factors *r* = 0.662, *p* = 0.019) in a bank of individual HLM (n = 12) (Fig 3B). Similarly, DMC-*O*-glucuronidation (G2) was strongly correlated with β-estradiol-3-*O*-glucuronidation (*r* = 0.711, *p* = 0.0096) (Fig 3C). Based on these results, UGT1A1 played a critical role in DMC-*O*-glucuronidation (G1 and G2).

### Concentration-dependent excretion of G1 and G2 in HeLa1A1 cells

**Fig 4:**
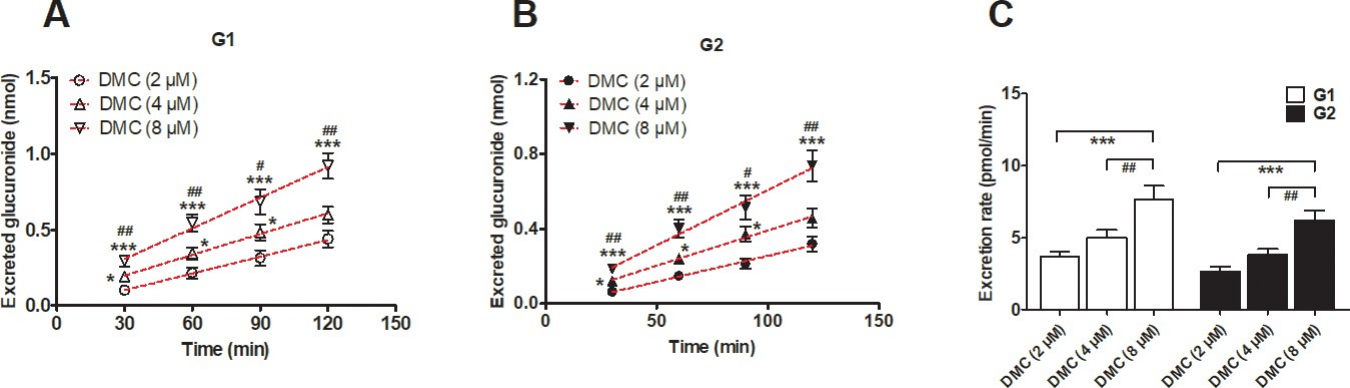
Concentration dependent excretions of demethoxycurcumin-*O*-glucuronides (G1 and G2) in HeLa1A1 cells. (A) Excreted demethoxycurcumin-*O*-glucuronides (G1) when treated with different concentrations of demethoxycurcumin (2, 4 and 8 μM); (B) Excreted demethoxycurcumin-*O*-glucuronides (G1) in extracellular solution at different concentrations of demethoxycurcumin (2, 4 and 8 μM); (C) Excreted rates of G1 and G2 at different concentrations (2, 4 and 8 μM); All experiments were performed in triplicate (n = 3). Data were expressed as mean ± SD (n = 3). *^, #^ *p* < 0.05, **^, ##^ *p* < 0.01 or ***^, ###^ *p* < 0.001 compared with that of demethoxycurcumin at 2 or 4 μM, respectively.

Since the K_m_ values of G1 and G2 were 4.5 to 5.0 μM and 2.7 to 3.8 μM, respectively (Table 1), DMC was applied at concentrations of 2, 4 and 8 μM to evaluate the excretion rates of G1 and G2. The excretion of G1 (Fig 4A) and G2 (Fig 4B) was markedly increased following incubation with the tested concentrations of DMC (2 to 8 μM). Furthermore, the rates of G1 and G2 excretion in the presence of various concentrations of DMC (2, 4 and 8 μM) showed significant differences (*p* < 0.05) (Fig 4C). Due to the similar K_m_ values of G1 and G2, DMC (4 μM) was used for the excretion assays.

### Effect of Ko143 and MK571 on the excretion of DMC-*O*-glucuronides

**Fig 5:**
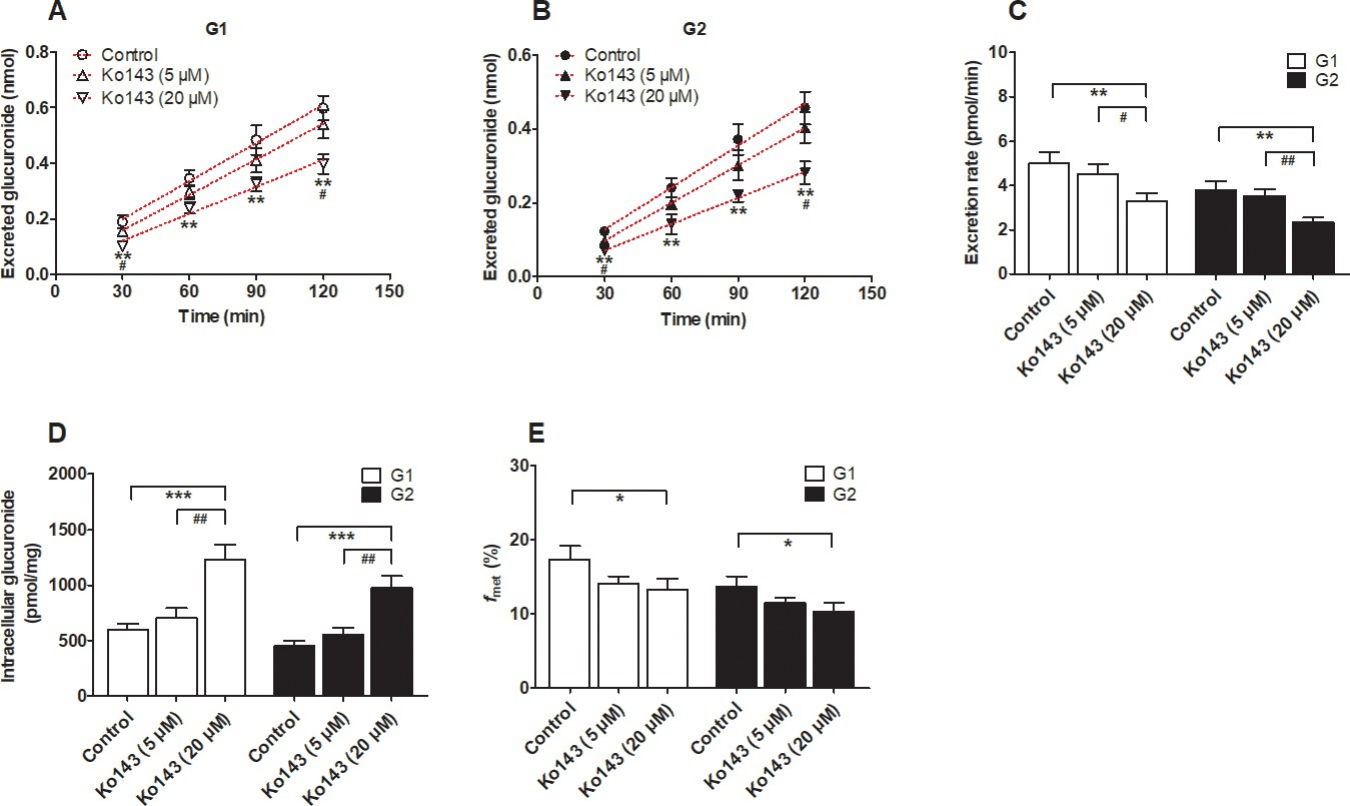
Effects of Ko143 on the formation and efflux excretion of demethoxycurcumin-*O*-glucuronides in HeLa1A1 cells. (A) Effects of Ko143 (5 and 20 μM) on the accumulation of demethoxycurcumin-*O*-glucuronides (G1) in extracellular medium; (B) Effects of Ko143 (5 and 20 μM) on demethoxycurcumin-*O*-glucuronides (G2) excretion in extracellular solutions; (C) Effects of Ko143 (5 and 20 μM) on the efflux excretion rates of G1 and G2; (D) Effects of Ko143 (5 and 20 μM) on the intracellular G1 and G2 levels; (E) Effects of Ko143 (5 and 20 μM) on the fraction metabolized (*f*_met_) of demethoxycurcumin; All experiments were performed in triplicate (n = 3). Data were presented as mean ± SD (n = 3). *^, #^ *p*<0.05, **^, ##^ *p*<0.01 and ***^, ###^ *p*<0.001 compared with that of control group of G1 or G2, respectively.

Ko143 and MK571 are well-accepted chemical inhibitors of the BCRP and MRP transporters, respectively [20–22]. As shown in Fig 5A and 5B, Ko143 (20 μM) resulted in a more efficient inhibition (24.8% to 45.4% for G1 and 24.3% to 40.9% for G2) of G1 and G2 excretion compared with Ko143 (5 μM) (9.8% to 18.6% for G1 and 7.2% to 19.8% for G2). When Ko143 (5 and 20 μM) was used, the intracellular levels of G1 and G2 were significantly elevated (17.6% to 104.8% for G1 and 24.0% to 117.6% for G2) (Fig 5D), whereas the excretion rates (9.8% to 33.7% for G1 and 7.2% to 38.6% for G2) (Fig 5C) and metabolized fractions (*f*_met_) (18.5% to 23.4% for G1 and 16.6% to 25.0% for G2) (Fig 5E) of G1 and G2 were both obviously decreased.

**Fig 6:**
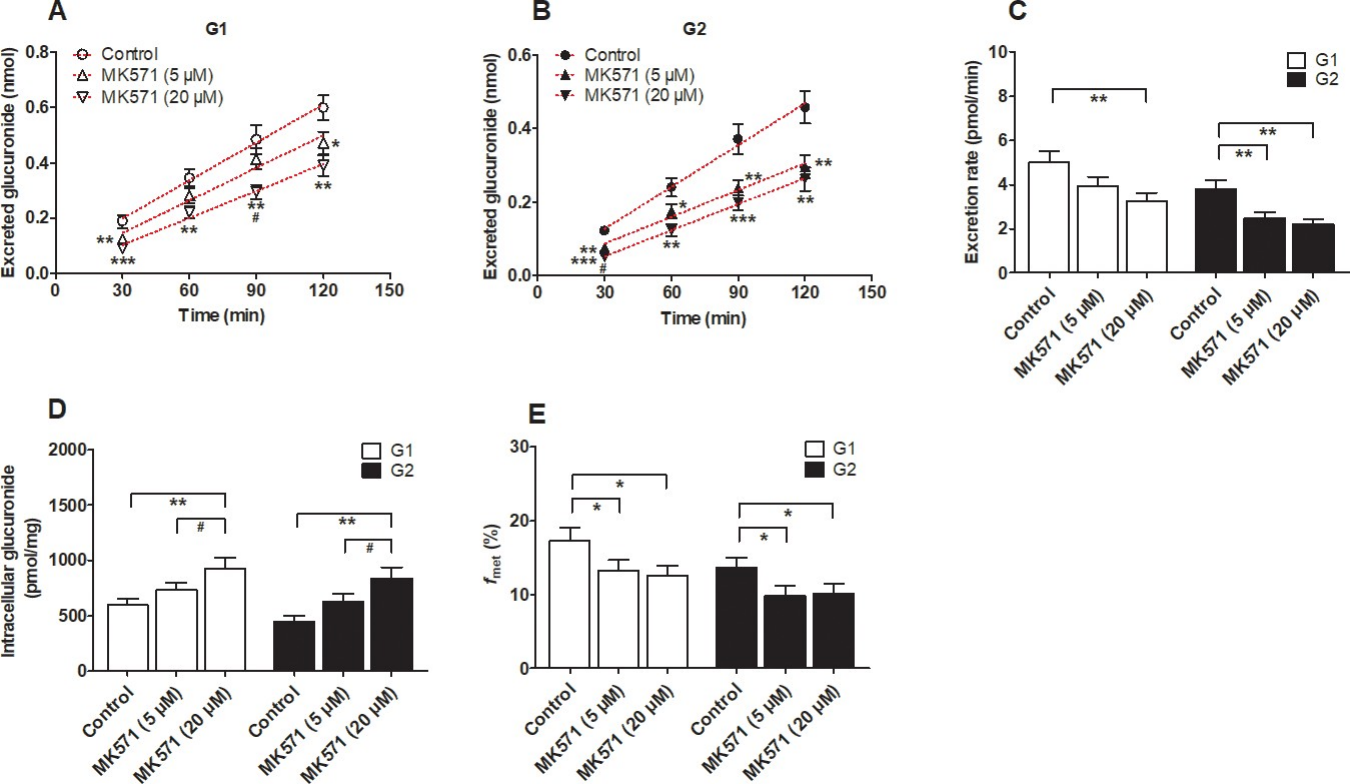
Transporter inhibitor MK571 inhibited the generation and excretion of demethoxycurcumin-*O*-glucuronides in HeLa1A1 cells. (A) MK571 (5 and 20 μM) inhibited the excretion of demethoxycurcumin-*O*-glucuronides (G1) in extracellular medium; (B) Inhibitory effects of MK571 (5 and 20 μM) on the excretion of demethoxycurcumin-*O*-glucuronides (G2) in extracellular solution; (C) MK571 (5 and 20 μM) decreased the efflux excretion rates of G1 and G2; (D) MK571 (5 and 20 μM) evaluated the intracellular levels of G1 and G2; (E) MK571 (5 and 20 μM) reduced the overall formation (*f*_met_) of G1 and G2 in HeLa1A1 cell; The concentration of demethoxycurcumin was 4 μM. All experiments were performed in triplicate (n = 3). Data were presented as mean ± SD (n = 3). *^, #^ *p* < 0.05, **^, ##^ *p* < 0.01 and ***^, ###^ *p* < 0.001 compared with that of control group of G1 or G2, respectively.

In addition, similar results were observed after treatment with MK571 (5 and 20 μM). MK571 (5 and 20 μM) clearly decreased the concentrations of excreted glucuronides (14.5% to 52.1% for G1, Fig 6A and 27.5% to 57.9% for G2, Fig 6B), the excretion rates (21.3% to 35.0% for G1 and 35.2% to 42.4% for G2) (Fig 6C) and the *f*_met_ values (23.5% to 27.0% for G1 and 25.9% to 28.0% for G2) (Fig 6E), following a significant increase in intracellular glucuronide concentrations (22.5% to 55.1% for G1 and 40.2% to 87.4% for G2) (Fig 6D). Therefore, BCRP and MRPs may play important roles in the excretion of DMC-*O*-glucuronides.

### Effects of Ko143 and MK571 on DMC-*O*-glucuronidation

**Fig 7:**
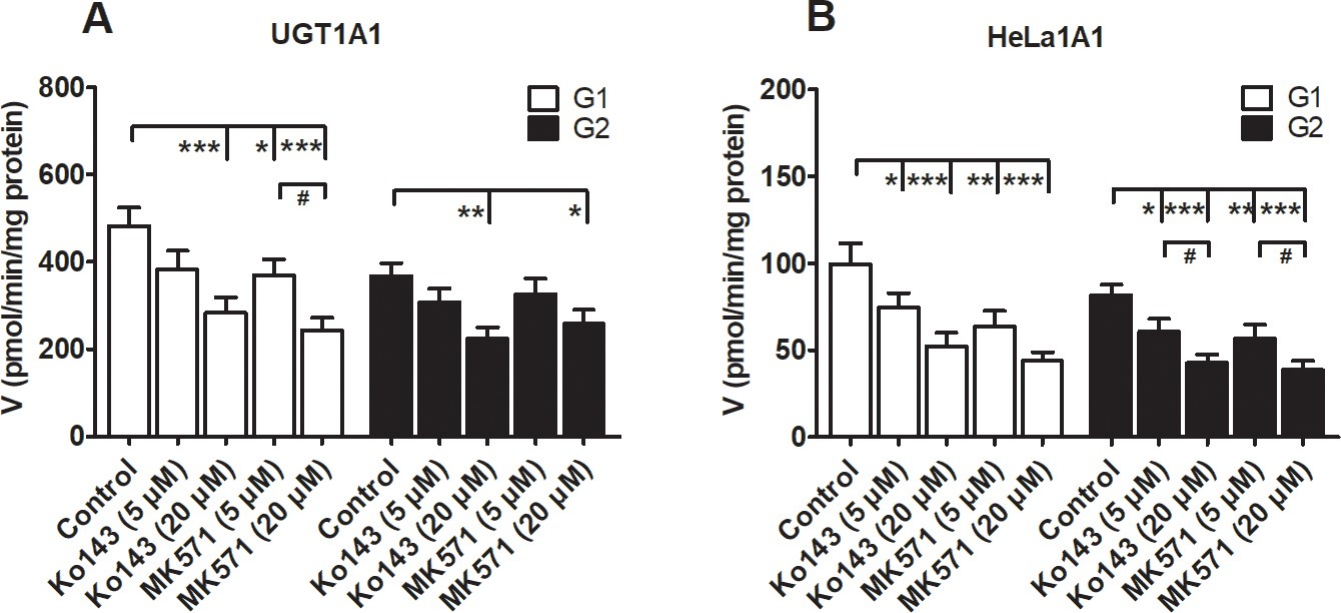
Inhibitory effects of chemical inhibitors Ko143 and MK571 on demethoxycurcumin-*O*-glucuronidation activity. (A) Inhibitory effects of chemical inhibitors on glucuronidation activity by UGT1A1; (B) Inhibitory effects of chemical inhibitors on glucuronidation activity by HeLa1A1 cell lysate. The concentration of demethoxycurcumin was 4 μM. All experiments were performed in triplicate (n = 3). Data were expressed as mean ± SD (n = 3). * *p* < 0.05, ** *p* < 0.01 and *** *p* < 0.001 compared with that of control group of G1 or G2, respectively. ^#^ *p* < 0.05, ^##^ *p* < 0.01 and ^###^ *p* < 0.001 compared with that of Ko143 (5 μM) or MK571 (5 μM), respectively.

As described previously, Ko143 and MK571 have been shown to exert inhibitory or stimulatory effects on the glucuronidation of drugs or natural products [21,22]. In our study, Ko143 and MK571 both exerted inhibitory effects on the formation of G1 and G2 induced by UGT1A1 (Fig 7A) and the HeLa1A1 cell lysate (Fig 7B). Ko143 (5 and 20 μM) displayed potent inhibition of the remaining UGT1A1 activity (58.8% to 79.4% for G1 and 60.9% to 83.5% for G2, Fig 7A) compared to the control values, while the remaining activity (50.6% to 76.7% for G1 and 70.2% to 88.9% for G2, Fig 7A) was also significantly decreased after treatment with MK571 (5 and 20 μM). Similar results were also obtained after Ko143 (52.7% to 75.1% for G1 and 52.3% to 74.4% for G2, Fig 7B) and MK571 (44.2% to 63.7% for G1 and 47.4% to 69.4% for G2, Fig 7B) were incubated with the HeLa1A1 cell lysate. Moreover, Ko143 and MK571 both exerted significant inhibitory effects on UGT1A1 activity, which also affected the validation of the functions of BCRP and MRPs in the excretion of DMC-*O*-glucuronides.

### Effect of the shRNA-mediated biological knock-down of BCRP and MRPs

The lentiviral constructs carrying shRNAs (BCRP-shRNA, MRP1-shRNA, MRP3-shRNA and MRP4-shRNA) were transfected to establish stable transporter knock-down cell lines [22]. Then, the protein levels of the silenced transporters were verified to be reduced by ≤ 50% after shRNA_BCRP and shRNA_MRPs plasmids were transiently transfected into HeLa1A1 cells.

**Fig 8:**
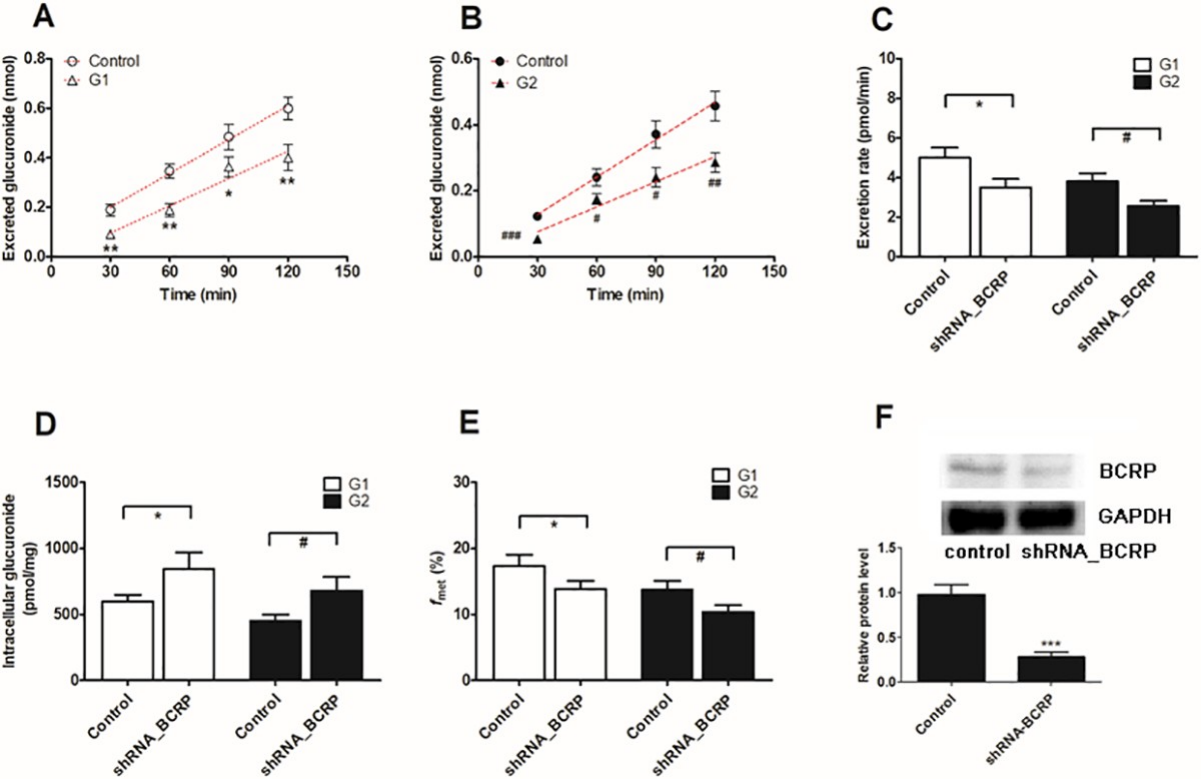
Effects of BCRP silencing on excretion rates and intracellular levels of demethoxycurcumin-*O*-glucuronides in HeLa1A1 cells. (A) Effects of excreted demethoxycurcumin-*O*-glucuronides (G1) in extracellular medium; (B) Effects of excreted demethoxycurcumin-*O*-glucuronides (G2) in extracellular medium; (C) Effects of the efflux excretion rates of G1 and G2; (D) Effects of the intracellular G1 and G2 levels; (E) Effects of the fraction metabolized (*f*_met_) of demethoxycurcumin; (F) Effects of gene silencing on the protein level of BCRP. The concentration of demethoxycurcumin was 4 μM. All experiments were performed in triplicate (n = 3). Data were presented as mean ± SD (n = 3). *^, #^ *p* < 0.05, **^, ##^ *p* < 0.01 and ***^, ###^ *p* < 0.001 compared with that of control group of G1 or G2, respectively.

BCRP silencing obviously reduced (29.2% to 51.5% for G1, Fig 8A, and 27.0% to 55.6% for G2, Fig 8B) the efflux transporter-mediated excretion of DMC-*O*-glucuronides (29.9% for G1 and 33.0% for G2) (Fig 8C) and significantly increased the intracellular levels of DMC-*O*-glucuronides (40.9% for G1 and 51.6% for G2) (Fig 8D). Meanwhile, the shRNA-mediated silencing of BCRP resulted in a marked decrease in the f_met_ value (20.1% for G1 and 24.5% for G2) (Fig 8E). The shRNA-mediated silencing of the BCRP gene resulted in obvious decreases in the levels of the BCRP protein (71%, *p* < 0.001, Fig 8F). Based on these results, the BCRP transporter played an important role in the excretion of DMC-*O*-glucuronides.

**Fig 9:**
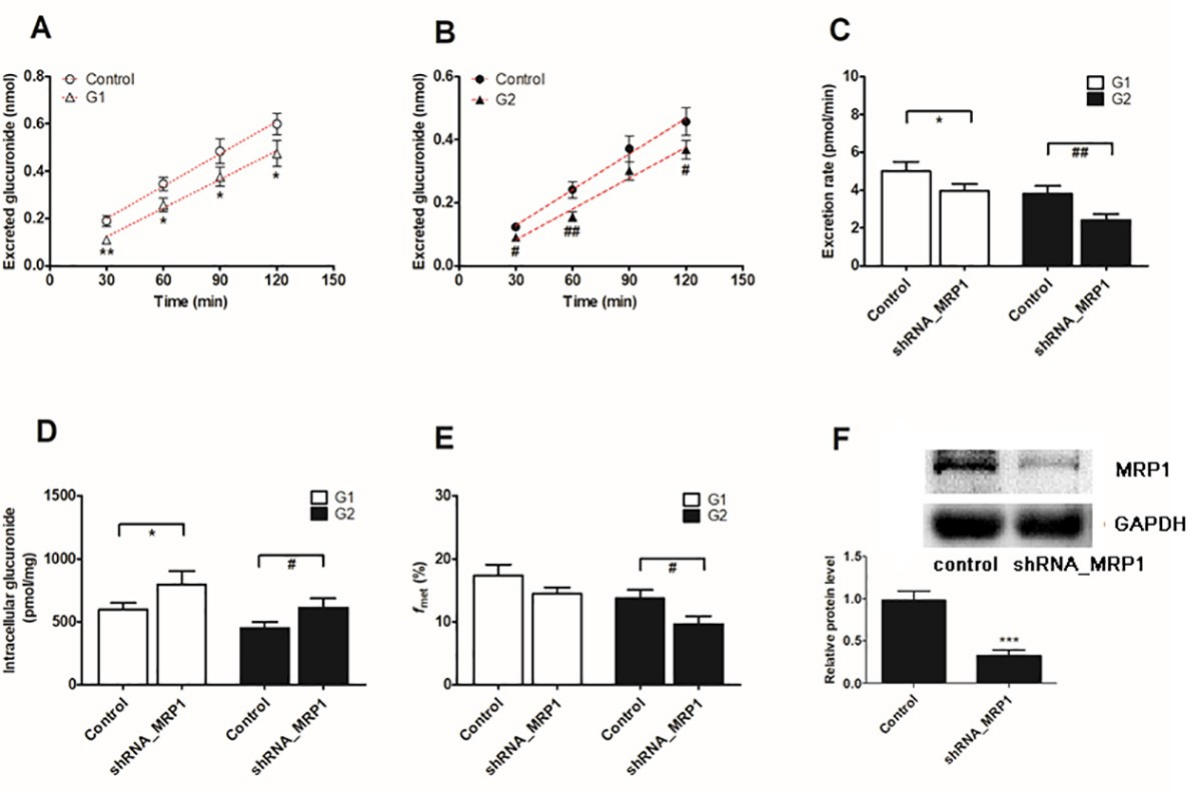
Effects of knock-down of MRP1 transporter on demethoxycurcumin-*O*-glucuronides excretion and cellular glucuronidation in HeLa1A1 cells. **(**A) Effects of accumlated demethoxycurcumin-*O*-glucuronides (G1) in extracellular medium; (B) Effects of excreted demethoxycurcumin-*O*-glucuronides (G2) in extracellular medium; (C) Effects of the excretion rates of G1 and G2; (D) Effects of the intracellular G1 and G2 levels; (E) Effects of *f*_met_ values of demethoxycurcumin; (F) Effects of gene silencing on the protein level of MRP1. The concentration of demethoxycurcumin was 4 μM. All experiments were performed in triplicate (n = 3). Data were expressed as mean ± SD (n = 3). *^, #^ *p* < 0.05, **^, ##^ *p* < 0.01 and ***^, ###^ *p* < 0.001 compared with that of control group of G1 or G2, respectively.

Similarly, knock-down of the MRP1 transporter also significantly decreased the excretion of DMC-*O*-glucuronides (20.8% to 42.3% for G1, Fig 9A, and 21.5% to 42.9% for G2, Fig 9B), the efflux excretion rates (20.8% for G1 and 36.9% for G2, Fig 9C) and the metabolized fractions (16.5% for G1 and 29.8% for G2, Fig 9E). In contrast, marked increases (33.1% for G1 and 36.1% for G2, Fig 9D) in the intracellular levels of DMC-*O*-glucuronides were observed after MRP1 silencing. Levels of the MRP1 protein in knock-down cells were reduced to approximately 33.7% of the level in the scrambled shRNA-transfected cells (Fig 9F).

**Fig 10:**
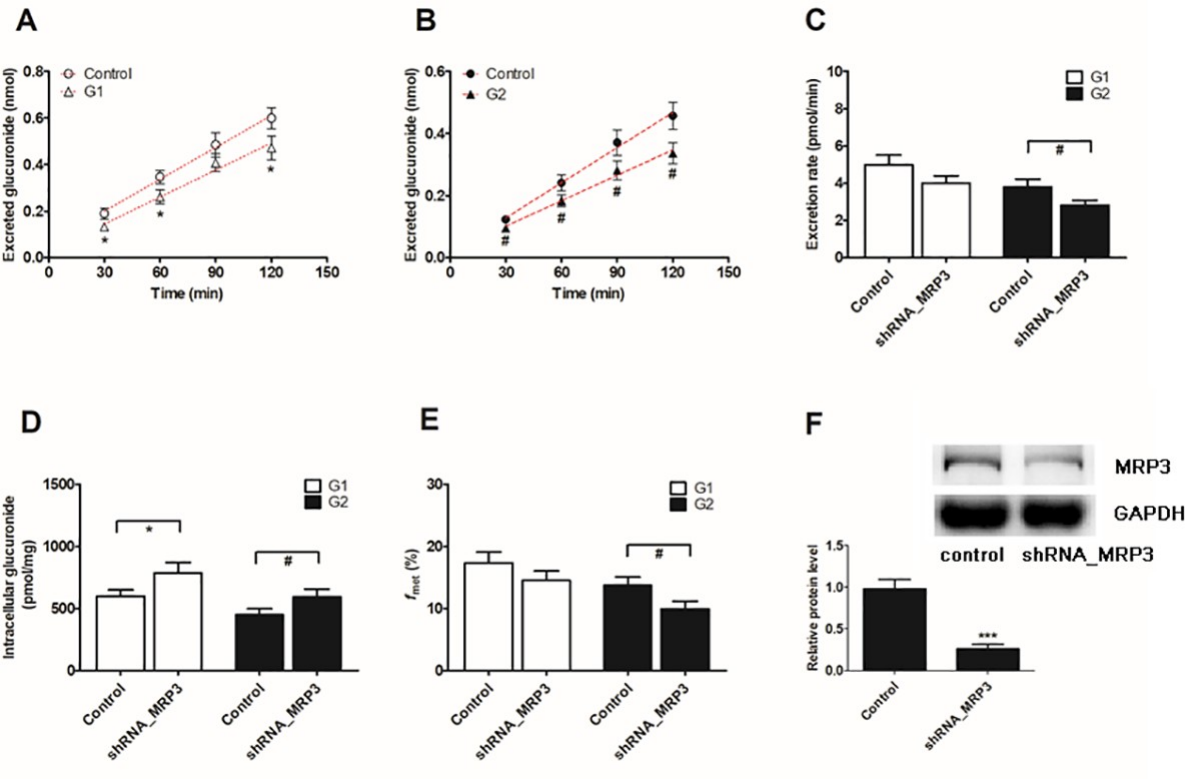
MRP3 silencing led to reduce excretion rates and increase intracellular demethoxycurcumin-*O*-glucuronides in HeLa1A1 cells. (A) MRP3 transporter silencing inhibited the accumlated demethoxycurcumin-*O*-glucuronides (G1) in extracellular medium; (B) MRP3 transporter silencing decreased the excreted demethoxycurcumin-*O*-glucuronides (G2) in extracellular solution; (C) MRP3 silencing significantly reduced the excretion rates of G1 and G2; (D) MRP3 silencing resulted in evaluation of intracellular G1 and G2 levels; (E) MRP3 silencing decreased total cellular glucuronidation (G1 ang G2) of demethoxycurcumin; (F) Effects of gene silencing on the protein level of MRP3. The concentration of demethoxycurcumin was 4 μM. All experiments were performed in triplicate (n = 3). Data were presented as mean ± SD. *^, #^ *p* < 0.05, **^, ##^ *p* < 0.01 and ***^, ###^ *p* < 0.001 compared with that of control group of G1 or G2, respectively.

In addition, the silencing of the MRP3 transporter significantly decreased protein expression (73.5%, *p* < 0.001, Fig 10F). Silencing also caused a moderate decrease in the excretion of DMC-*O*-glucuronides (10.1% to 29.9% for G1, Fig 10A, and 22.2% to 26.4% for G2, Fig 10B) and in the efflux excretion clearance (19.7% for G1 and 26.4% for G2, Fig 10C), whereas a marked increase in the intracellular levels of DMC-*O*-glucuronides was observed (31.5% for G1 and 32.1% for G2, Fig 10D). In addition, the *f*_met_ values were significantly decreased (16.2% for G1 and 27.9% for G2, Fig 10E) following the partial knock-down of the MRP3 transporter.

**Fig 11:**
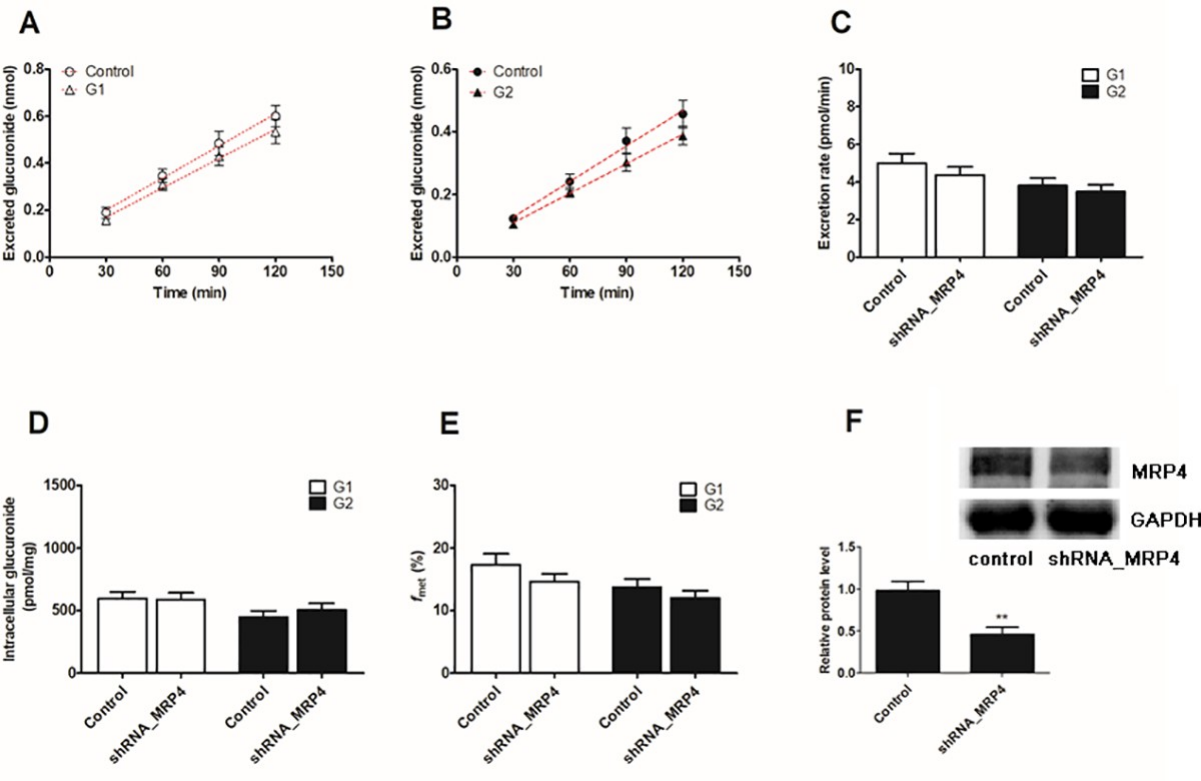
Effects of shRNA-mediated MRP4 transporter knock-down on demethoxycurcumin-*O*-glucuronides excretion and cellular glucuronidation in HeLa1A1 cells. (A) MRP4 knock-down decreased the excreted demethoxycurcumin-*O*-glucuronides (G1); (B) MRP4 knock-down reduced the accumlated demethoxycurcumin-*O*-glucuronides (G2) in extracellular medium; (C) MRP4 knock-down inhibited the excretion rates of G1 and G2; (D) The intracellular G1 and G2 levels were evaluated when MRP4 transporter was knock-down; (E) MRP4 knock-down resulted in decrease of overall cellular glucuronidation (G1 and G2) of demethoxycurcumin; (F) Effects of gene silencing on the protein level of MRP4. The concentration of demethoxycurcumin was 4 μM. All experiments were performed in triplicate (n = 3). Data were expressed as mean ± SD (n = 3). *^, #^ *p* < 0.05, **^, ##^ *p* < 0.01 and ***^, ###^ *p* < 0.001 compared with that of control group of G1 or G2, respectively.

However, almost no changes (*p* > 0.05) in the levels of excreted glucuronides (Fig 11A and 11B), efflux excretion rates (Fig 11C), intracellular levels of DMC-*O*-glucuronides (Fig 11D) and *f*_met_ values (Fig 11E) were observed after the silencing of MRP4. The levels of the MRP4 protein in HeLa1A1-MRP4-shRNA cells were approximately 46.9% of the levels in the scrambled shRNA-transfected cells (Fig 11F). Thus, the changes in the excretion of DMC-*O*-glucuronides was primarily mediated by the reduced expression of the BCRP, MRP1 and MRP3 transporters.

## Discussion

DMC is a safe and natural food-coloring additive with anti-inflammatory, antioxidant, and anticarcinogenic activities [2–4]. However, poor absorption in the plasma [7,8] and extensive phase I and phase II metabolism in the liver and intestine [12–14] lead to the low bioavailability of DMC in animals and humans, which limits its use as a therapeutic agent. DMC-*O*-glucuronides were identified as the most abundant metabolites of DMC due to a high concentration in rat plasma (approximately 50 nM) [13]. UGT1A1 is primarily responsible for glucuronidation [18], consistent with our results (Figs 2 and 3). In addition, the probe substrate β-estradiol [24] was used to perform validate the function of UGT1A1 in the previously established HeLa1A1 cells [22]. UGT1A1 has traditionally been thought to be primarily expressed in the liver and intestine [24]. Based on these findings, DMC-*O*-glucuronidation in the human liver and intestine should not be underestimated when determining the oral bioavailability of DMC.

On the other hand, DMC also exhibited inhibitory or stimulatory effects on certain drug-metabolizing enzymes after being metabolized by these enzymes. For example, DMC inhibited certain subtypes of human cytochrome P450 (CYP) enzymes more potently, including CYP2C9 (IC_50_ = 1.4 μM), CYP3A4 (IC_50_ = 7.0 μM) and CYP1A2 (IC_50_ = 34.0 μM) [31]. In addition, a pronounced inhibitory effect of DMC was observed, with IC_50_ values of 31.5, 8.8, 1.7 and 13.9 μM for CYP3As, 2C9, SULTs and UGTs, respectively [32]. In contrast, DMC was activated phase II enzymes (GSTs, UGTs, epoxide hydrolase, and other enzymes) (circular dichroism, CD value = 9.5 μM), as evidenced by its ability to increase the enzymatic activity of quinone reductase (QR) in murine hepatoma cells [33]. Because DMC exerts potent and broad-spectrum inhibitory or stimulatory effects on human drug-metabolizing enzymes, much caution should be exercised when high-dose DMC or DMC-containing herbal medicines are co-administered with the substrates (particularly clinical drugs) of the corresponding drug-metabolizing enzymes.

In addition, the use of chemical inhibitors to identify the efflux transporters that are responsible for glucuronide excretion should be performed with caution due to the potential of these inhibitors to modify the glucuronidation activity. In the present study, Ko143 exerted obvious inhibitory effects not only on the excretion of DMC-*O*-glucuronides (Fig 5) but also on the DMC-*O*-glucuronidation activity mediated by UGT1A1 (Fig 7A) and the HeLa1A1 cell lysate (Fig 7B). The inhibition of DMC-*O*-glucuronide excretion was primarily attributed to the significant inhibition of the BCRP transporter by Ko143 (IC_50_ = 23 nM) [34]. However, the inhibitory effect of Ko143 on the DMC-*O*-glucuronidation activity (Fig 7) would substantially affect analyses of the function of the BCRP transporter in the excretion of DMC-*O*-glucuronides (Fig 5). Similar results were also observed when MK571, a pan-MRP inhibitor, was used (Figs 6 and 7). Notably, these findings are consistent with previous studies [21,22]. Therefore, chemical assays are usually considered an auxiliary approach to identify the active efflux transporters.

Biological knock-down assays, including the shRNA-mediated silencing of BCRP or MRPs, are considered a more reliable approach to evaluate the roles of efflux transporters [29]. In the present study, levels of the BCRP or MRP proteins were significantly decreased to 20% to 40% of the levels in the control group, according to the western blotting assays, which was also consistent with previous studies [21,22]. Furthermore, the shRNA-mediated silencing of the BCRP (Fig 8), MRP1 (Fig 9) and MRP3 (Fig 10) genes all led to an obvious reduction in the levels of excreted DMC-*O*-glucuronides, excretion rates and metabolized fractions, and a significant increase in the intracellular levels of DMC-*O*-glucuronides. In contrast, these alterations were not observed when MRP4 was partially silenced (Fig 11). Based on these results, DMC was subjected to extensive glucuronidation by UGT1A1 (Figs 2 and 3), and BCRP, MRP1 and MRP3 were the main transporters contributing to the disposition and excretion of DMC-*O*-glucuronides, consistent with the proposed efflux mechanism of bisdemethoxycurcumin, a demethoxylated derivative of DMC [35].

However, this HeLa1A1 cell model had a serious limitation regarding the analysis of the functions of MRP2 and MRP5 in the efflux excretion of drugs due to the absence of these two transporters in HeLa cells [36]. Traditionally, MRP2 has been thought to be the most highly expressed transporter in the liver, where it facilitates the elimination of bilirubin glucuronides and positively charged drugs and conjugates the bile [37]. Furthermore, MRP5 has not been as extensively studied as other drug transporters; thus, information on its potential roles in drug disposition and excretion or its toxicity is limited [38]. For an evaluation of MRP2 and MRP5 functions, previous studies have provided practical and effective examples in MDCKII-MRP2-UGT1A1 cells [39] and MDCKII-OATP1B1-UGT1A1-MRP2 cells [40]. Currently, the most commonly used method is to stably transfect HeLa cells, MDCKII cells, HEK cells or Caco-2 cells with the established plasmids carrying the cDNAs encoding MRP2, MRP5, other drug metabolizing enzymes, or uptake or efflux transporters. This method is also convenient for investigating the roles of drug-metabolizing enzymes and transporters in the disposition of drugs.

DMC was also capable of inhibiting the activities of human intestinal P-glycoprotein (P-gp) [41]. To date, the effects of DMC on other transporters remain unknown. However, curcumin, the methoxylated form of DMC, was reported to exert inhibitory effects on the BCRP transporter (K_i_ = 0.70 μM) [42], which contributed to the regulatory effect of aryl hydrocarbon receptor [43]. This inhibition of BCRP exhibited pharmacokinetic interactions (particularly the AUC_0-t_ values) when clinical drugs and curcumin were co-administered orally [44]. In addition, curcumin clearly inhibited both MRP1- and MRP2-mediated transport with IC_50_ values of 15 and 5 μM, respectively, which also affected the disposition of other xenobiotics [45]. Therefore, the effects of DMC on these efflux transporters warrant further exploration. The implication of these findings is that the regular consumption of DMC and DMC-containing foods or herbs potentially produces food- or herb-drug interactions; thus, avoiding the consumption of drugs that are substrates of P-gp, BCRP and MRPs may be a prudent choice.

Although efflux transporters are primarily responsible for the elimination of glucuronides, their possible impacts on disposition of the parent drug have received attention, likely because glucuronides are generally pharmacologically inactive [19]. Actually, significant interplay has been observed between glucuronide formation and excretion, which is also called futile recycling [20] or glucuronidation-transport interplay [21,22]. Without futile recycling, glucuronide formation would be independent of its downstream process, excretion, and thus the impact of metabolite excretion on its formation would be impossible [19]. Simply stated, glucuronides can be hydrolyzed back to the parent drug by β-glucuronidases in cells [21,22], which alters the systemic exposure of the parent drug. Thus, the metabolized fraction (*f*_met_) is regarded as the more appropriate parameter to reflect the extent of drug metabolism in intact cells in the presence of a transporter-enzyme interplay [29]. Therefore, the BCRP (Fig 8) transporter was the most important contributor to DMC-*O*-glucuronide (G1) disposition, whereas BCRP (Fig 8), MRP1 (Fig 9) and MRP3 (Fig 10) were primarily responsible for the excretion of DMC-*O*-glucuronides (G2).

Recently, gene polymorphisms have received increasing attention in the clinic because physicians have the goal of administering individualized, precise medication. Human UGT1A1 not only catalyzes the glucuronidation of approximately 15% of marketed drugs (irinotecan, cyproheptadine, morphine, and other drugs), but this enzyme maintains a stable balance of endogenous substances (bilirubin, bile acids, estrogen, and other substances) in the human body [46]. Nonetheless, we only focused on wild-type UGT1A1 in this study. In reality, functional UGT1A1 polymorphisms have been systematically identified, and newly identified variants have been provided in an updated list (www.pharmacogenomics.pha.ulaval.ca). Accordingly, the distribution frequency of UGT1A1*6 and UGT1A1*28 in the Chinese population were shown to be 23% and less than 10%, respectively, and individuals with these variants are more prone to suffer adverse reactions when treated with irinotecan and SN-38 (www.PharmGKB.org). On the other hand, an abnormality or deficiency in UGT1A1 *in vivo* is strongly correlated with certain diseases (Gilbert syndrome, Crigler-Najjar syndrome, and hyperbilirubinemia), the toxicity of drugs, and the precise therapeutic profile [47]. Hence, an investigation of the disposition of DMC, other drugs or natural bioactive compounds has clinical significance.

Similarly, numerous single-nucleotide polymorphisms (SNPs) in the BCRP and MRP transporter genes have been identified. The nonsynonymous SNP e5/C421A was shown to be associated with lower BCRP expression, as the protein is less stable and has reduced plasma membrane localization [48]. This variant led to the an increase (3.2-fold) in the AUC of orally administered salazosulfapyridine (SASP) following a curcumin pretreatment in subjects with the ATP-binding cassette sub-family G member 2 (ABCG2) 421CC genotype, which was comparable to the clinical phenotype observed in subjects with the ABCG2 421CA genotype (2.1- to 3.5-fold) [24]. In addition, genetic variations in ABBC1 were shown to be associated with the severity of hematological adverse events in 5-fluorouracil (5-FU)-, epirubicin- or cyclophosphamide-treated patients with breast cancer [49]. Carriers of the MRP3 189A>T allele also present higher plasma levels of methotrexate, and gene reporter assays revealed increased promoter activity for the A-189 allele, indicating the increased efflux activity of MRP3 [50]. The variants of efflux transporters result in increased or decreased plasma exposure of clinical therapeutic drugs; therefore, dose adjustments of these drugs should be recommended for carriers of certain variants in the clinic.

## Conclusions

In conclusion, the engineered HeLa1A1 cells described herein successfully expressed the UGT1A1 protein, which catalyzed the glucuronidation of β-estradiol and DMC (Fig 1) and further excreted the glucuronides in a concentration-dependent manner, as shown in Fig 4. In addition, DMC-*O*-glucuronidation followed the classical Michaelis-Menten kinetics (Fig 2). Moreover, Ko143 (Fig 5) and MK571 (Fig 6) both significantly reduced the cellular excretion of DMC-*O*-glucuronides. Moreover, the presence of MRP4 did not significantly alter the efflux excretion of DMC-*O*-glucuronides (Fig 11); hence, the roles of BCRP, MRP1 and MRP3 remain to be investigated. Remarkably, BCRP (Fig 8) was shown to play a critical role in the disposition and excretion of DMC-*O*-glucuronides, whereas the contributions of MRP1 (Fig 9) and MRP3 (Fig 10) were moderate. Based on these results, extensive glucuronidation and metabolism by UGT1A1 and efflux transport by BCRP, MRP1 and MRP3 have the greatest contributions to the poor bioavailability of DMC. In addition, this study reported an engineered HeLa1A1 cell model to manipulate the oral bioavailability of other therapeutic drugs or relevant natural products and investigate the corresponding glucuronidation-transport interplay at the cellular level.
